# Platelet-Rich Plasma May Offer a New Hope in Suppressed Wound Healing When Compared to Mesenchymal Stem Cells

**DOI:** 10.3390/jcm7060143

**Published:** 2018-06-08

**Authors:** Oktay Aydin, Gökhan Karaca, Faruk Pehlivanli, Canan Altunkaya, Hafize Uzun, Hüseyin Özden, Gülçin Aydin, İbrahim Tayfun Şahiner, Mehmet Niyaz, Osman Güler

**Affiliations:** 1 Department of General Surgery, School of Medicine, Kirikkale University, 71450 Kirikkale, Turkey; gokhankaracaa@gmail.com (G.K.); drfapeh@hotmail.com (F.P.); oguler59@hotmail.com (O.G.); 2 Department of Pathology, School of Medicine, Kirikkale University, 71450 Kirikkale, Turkey; altunkayacanan@gmail.com; 3 Department of Medical Biochemistry, Cerrahpaşa Medical Faculty, İstanbul University, 34452 Istanbul, Turkey; hafizeuzun@gmail.com; 4 Department of General Surgery, Çorum Alaca State Hospital, 19600 Çorum, Turkey; drhuseyinozden@gmail.com; 5 Department of Anesthesiology and Reanimation, Kirikkale University, School of Medicine, 71450 Kirikkale, Turkey; drgulcinaydin@yahoo.com; 6 Department of General Surgery, School of Medicine, Hitit University, 19030 Çorum, Turkey; tayfunsahiner@gmail.com; 7 Department of Cardiovascular Surgery, Bartin State Hospital, 74100 Bartin, Turkey; mehmetniyaz@gmail.com

**Keywords:** platelet-rich plasma, mesenchymal stem cell, wound healing

## Abstract

Background: The present study investigated the effectiveness of platelet-rich plasma (PRP) and mesenchymal stem cells (MSCs) in wound healing suppressed by corticosteroid in rats. Methods: Forty rats were separated into four groups. To disrupt the wound-healing processes, intraperitoneal single dose 10 mg/kg methylprednisolone was administered to all rats with the exception of Sham-S group. Then, full-thickness incision was performed to the abdominal skin of all animals, and PRP or MSCs were applied to the incision line except the Sham-S and Sham-M group animals. Ten days later, all animals were sacrificed to investigate: tissue collagenization, inflammation, and re-epithelialization grades histopathologically; and tissue hydroxyproline (HP), interleukin-1β (IL-1β), tumor necrosis factor-α levels biochemically. Results: Collagenization (*p* = 0.003) and inflammation grade (*p* = 0.002) values were higher in PR group. Tissue HP level value was found to be high in MC group (*p* < 0.001). Tissue IL-1β level value of Sham-M group was lower than those of other groups (*p* < 0.001). Conclusions: This preliminary study revealed that PRP could improve the histopathological grades in wound healing which was suppressed by corticosteroid in rats, while MSCs could show their therapeutic effects via biochemical route. These positive effects were more salient in PR group.

## 1. Introduction

Wound healing is an interwoven and continuous set of physiological and biochemical events that includes inflammation, proliferation, and remodeling [1,2].

Platelet-rich plasma (PRP), an autologous plasma portion that is enriched with the platelets three to five times more than the baseline plasma levels, contains high concentrations of autogenous growth factors (e.g., vascular endothelial growth factor (VEGF), insulin-like growth factor (IGF), platelet derived growth factor (PDGF), transforming growth factor-1β (TGF-1β)), certain proteins and peptides (such as fibrinogen, fibronectin, osteonectin, osteocalcin, vitronectin, thrombospondin), and certain chemokines and cytokines (such as IL-1, platelet factor 4) [1,3,4,5]. The several types of PRP have long been used in many clinics including orthopedics, dentistry, neurosurgery, ophthalmology, urology, cosmetic, mentoplasty and facial surgery due to their enhancing effects on wound healing, cellular mitogenesis, osteogenesis and angiogenesis [6,7,8,9,10]. Epithelial differentiation and organization are more uniform in the dermal matrix of wounds treated with PRP, which accelerates epithelization by increasing vascular growth, fibroblast proliferation and collagen production [5,11].

However, mesenchymal stem cells (MSCs)—which have a self-renewal capacity and the ability to differentiate into more mature cells and different tissue types by asymmetric replication—play a central role in wound healing, and accelerate cell proliferation, granulation tissue formation, and neovascularization by reducing cytokine release in injured tissue while they migrate, concentrate and differentiate in the damaged tissue [12,13,14,15]. Furthermore, after the local administration to the wound area they may exhibit cell-protective activity in the presence of appropriate inflammatory mediators through the direct effects or paracrine effects of the growth hormones [16,17,18,19,20,21,22].

In contrast, corticosteroids adversely affect wound-healing processes at multiple stages such as suppressing the inflammatory cell migration, fibroblast proliferation, capillary regeneration, epithelial migration, and type I collagen synthesis [23]. Therefore, the application of corticosteroids decreases inflammation while disrupting collagen synthesis and reducing wound contraction [24].

The aim of this preliminary experimental study was to investigate the possible positive and therapeutic effects of PRP and MSCs on wound healing suppressed by corticosteroid in rats.

## 2. Materials and Methods

### 2.1. Materials

The study was conducted according to the rules and procedures stipulated by the Local Ethics Committee and after the necessary permission had been obtained (Decision no: 2014/40, dated: 27th February 2014).

In this study, 5 of 48 male Wistar albino rats (each weighing 250–280 g) that were not included in the study groups were sacrificed by totally removing of blood, which was used for preparation of the PRP. Three other rats that were not included into the study groups were used to prepare the MSCs from the adipose tissue taken from the lower abdomen. The remaining rats (*n* = 40) were randomly divided into four groups as follows:

- Sham-S group (vehicle group on which surgery was performed, with no experimental biological material being infused; *n* = 10)
- Sham-M group (vehicle group on which surgery was performed following an intraperitoneal injection of corticosteroid, with no other experimental biological material being administered; *n* = 10)
- PR group (surgery was performed following an intraperitoneal injection of corticosteroid, and then PRP was administered into the wound; *n* = 10)
- MC group (surgery was performed following an intraperitoneal injection of corticosteroid, and then MSCs were administered into the wound; *n* = 10)

To obtain the PRP, venous blood taken from the vena cava inferior of the donor rats (*n* = 5) was collected into tubes comprising 3.2% sodium citrate (Merck, Darmstadt, Germany), and centrifuged at 400 G for 10 min to obtain a supernatant. The collected supernatant was re-centrifuged at 800 G for 10 min. After the centrifuge, the upper two-thirds of the plasma was removed, and the lower one-third was kept as PRP.

MSCs were prepared from the adipose tissue taken from the lower abdomen of the donor rats (*n* = 3) under sterile conditions in the Department of Biology at Hacettepe University.

### 2.2. Surgery

First, with the exception of animals in the Sham-S group, a single dose of 10 mg/kg methylprednisolone (Prednol-L, Mustafa Nevzat, Istanbul, Turkey) was injected into each rat intraperitoneally to disrupt the wound-healing processes. Second, all the rats were anesthetized by intraperitoneal injections of 12 mg/kg ketamine hydrochloric acid (HCl) (Ketalar^®^; Pfizer Inc., New York, NY, USA), and 80 mg/kg xylazine HCl (Rompun^®^ %2; Bayer HealthCare AG, Leverkusen, Germany), and a 4 cm full-thickness skin incision was made to the abdominal front wall. Then, experimental biological materials (PRP or MSCs) were slowly applied into the incision line before closing the wound. PRP, administered as a single dose, was applied to the wound in the amount of 1 mL. The MSCs, administered as a single dose, were also applied in the amount of 1 mL, containing 3 × 10^6^ MSCs. Ten days later, all the rats were sacrificed by administering an overdose of anesthetic agents and the old skin incision line with its surrounding tissue was removed, with half being immersed into 10% formalin, and the remaining half being immediately refrigerated at −30 °C.

### 2.3. Histopathological Analysis

**Figure 1:**
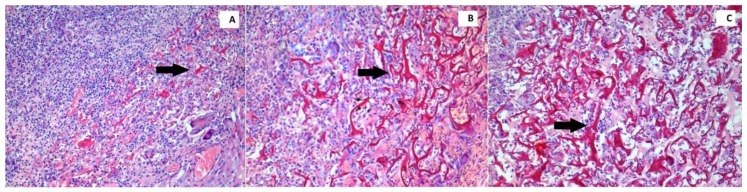
Histopathological images demonstrate the grade 1 (**A**), grade 2 (**B**), and grade 3 (**C**) collagenization density in the wound (H&E, ×100).

**Figure 2:**
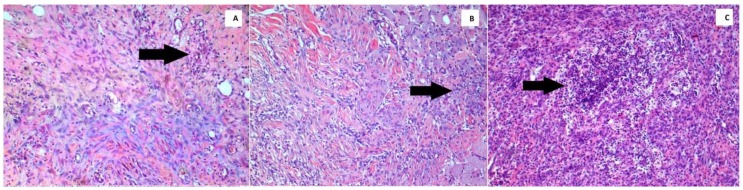
Histopathological images demonstrate the grade 1 (**A**), grade 2 (**B**), and grade 3 (**C**) inflammation severities in the wound (H&E, ×100).

**Figure 3:**
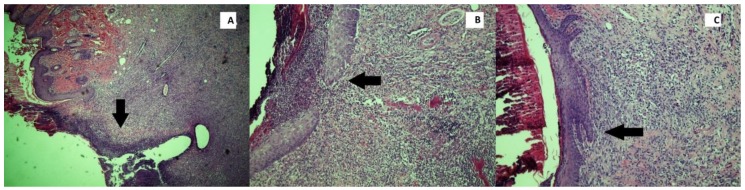
Histopathological images demonstrate the grade 0 (**A**), grade 1 (**B**), and grade 2 (**C**) of the reepithelization processes in the wound (H&E, ×100).

All specimens were fixed with 4% paraformaldehyde in phosphate-buffered saline (pH = 7.4). Subsequently, the tissues were embedded in paraffin blocks and sections 4–5 μm in thickness were taken from these tissues. The sections were stained with hematoxylin and eosin (H&E) and placed under a light microscope (Olympus BX51; Olympus, Tokyo, Japan), where the wound-healing parameters collagenization (Figure 1), inflammation (Figure 2) and reepithelialization (Figure 3), were evaluated and scaled by a pathologist who was blinded to the study groups and experimental agents (Table 1) [25]. All inflammatory cells were counted in areas per section of the wounded skin tissue, and in each group the average number per rat was calculated to construct and score the inflammatory grade values.

### 2.4. Biochemical Analysis

Samples were thawed, and an enzyme immunoassay kit (Eastbiopharm, Hangzhou, China) was used for quantitative measurement of the tissue samples. To demonstrate the collagenization level in the wound healing, the tissue hydroxyproline (HP) level was measured [26]. Optical density was read on a standard automated plate reader at 450 nm (PerkinElmer, 1420 Victor3, Waltham, MA, USA), and tissue HP (Rat Hydroxyproline Elisa Kit, Eastbiopharm Co., Ltd., Hangzhou Assay; Range: 20–1000 Ng/L), interleukin-1β (IL-1β) (Rat IL-1β Elisa Kit, Eastbiopharm Co., Ltd., Hangzhou Assay; Range: 20–800 Ng/L), tumor necrosis factor-α (TNF-α) (Rat TNF Alpha Elisa Kit Eastbiopharm Co., Ltd., Hangzhou Assay; Range: 5–1000 ng/L) levels were measured using the enzyme linked immunoabsorbent assay (ELISA).

### 2.5. Statistical Analysis

Descriptive data were expressed in mean or median values. Tissue HP and IL-1β level values—which were distributed normally among the groups—were analyzed using the One Way Analysis of Variance (ANOVA). A binary comparison was conducted using the Tukey Multiple Comparisons test and a p-value of <0.05 was considered to be statistically significant.

Collagenization, inflammation, reepithelization grade values and tissue TNF-α level values which were not normally distributed among the groups were analyzed using the Kruskal-Wallis test. The binary comparison was made using the Mann-Whitney U test and Bonferroni Correction test. A *p*-value of <0.0083 was considered to be statistically significant.

## 3. Results

### 3.1. Histopathological Examination

**Figure 4:**
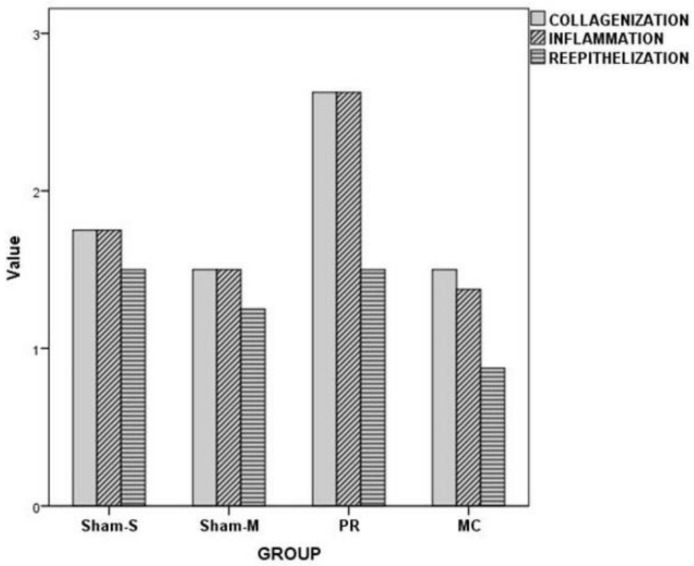
Histopathological grading results of all groups.

The collagenization (*X*^2^ = 13.89, *p* = 0.003) and inflammation (*X*^2^ = 14.79, *p* = 0.002) grade values were statistically different among the groups. However, the reepithelialization score values were not different among the groups (Table 2 and Table 3, Figure 4).

The collagenization level value of the PR group was higher than Sham-M (*Z* = −2.92, *p* = 0.003), Sham-S (*Z* = −2.71, *p* = 0.007), and MC (*Z* = −2.92, *p* = 0.003) group values. The inflammation grade value of the PR group was higher than Sham-M (*Z* = −2.92, *p* = 0.003), Sham-S (*Z* = −2.71, *p* = 0.007), and MC (*Z* = −3.06, *p* = 0.002) group values (Table 4).

### 3.2. Biochemical Examination

**Figure 5:**
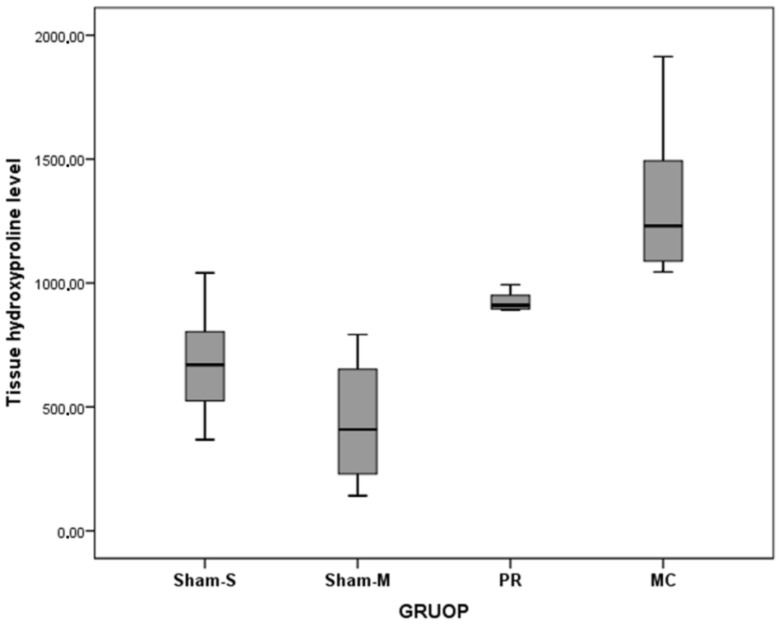
Tissue hydroxyproline level values of all groups.

**Figure 6:**
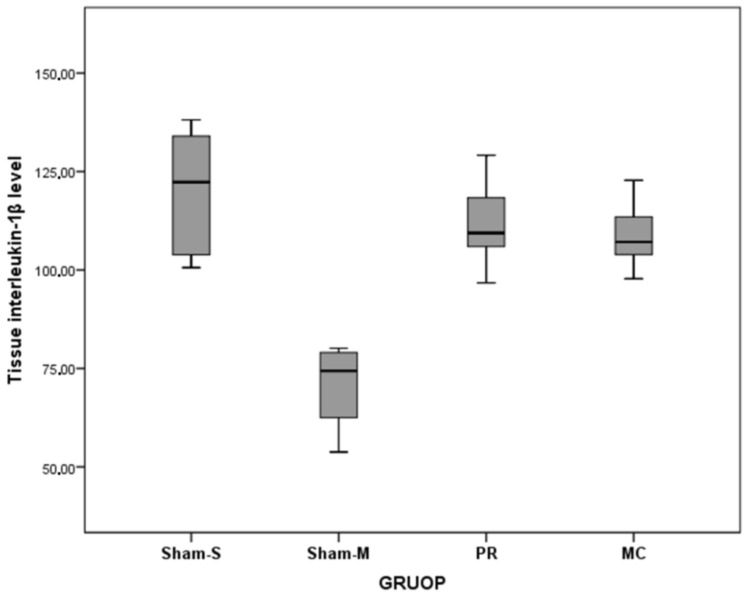
Tissue interleukin-1β level values of all groups.

**Figure 7:**
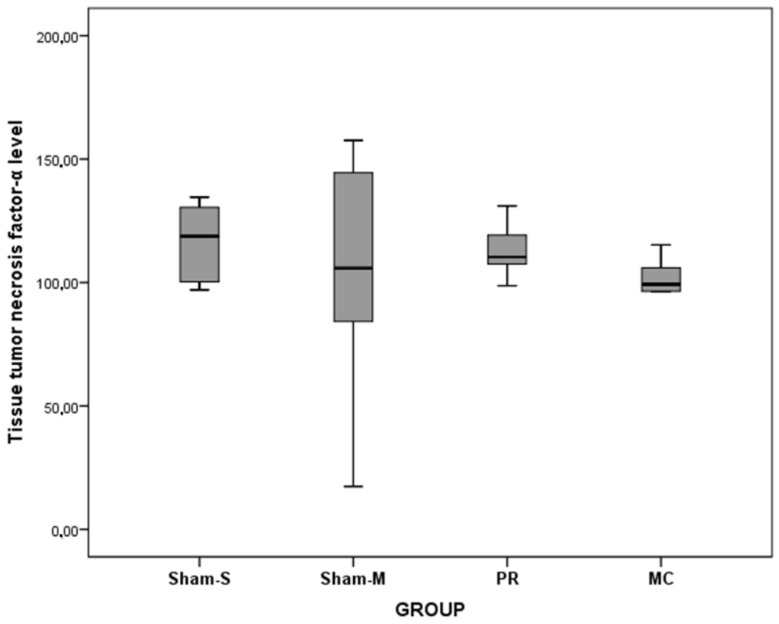
Tissue tumor necrosis factor-α level values of all groups.

The tissue HP (*F* = 22.34, *p* < 0.001) and IL1β (*F* = 8.64, *p* < 0.001) level values were statistically different among the groups. However, TNF-α level values were not different among the groups (Table 2 and Table 3, Figure 5, Figure 6 and Figure 7). The values of the tissue HP level of the MC group were higher than Sham-S (MD = −647.17, *p* < 0.001) and Sham-M (MD = −883.33, *p* < 0.001) groups. The tissue HP level value of the PR group was higher than Sham-M (MD = −460.82, *p* = 0.002) group. The tissue IL-1β level value of the Sham-M group was lower than Sham-S (MD = −41.17, *p* < 0.001), PR (MD = −32.94, *p* = 0.004), and MC (MD = −29.99, *p* = 0.008) groups (Table 4).

## 4. Discussion

Collagen is primarily responsible for the wound strength and is important for wound healing. However, different collagen types have varying amounts of HP (for example, type III contains more HP residues than those contained in the alpha-chains of type I) and it has been accepted that HP level is associated with the collagen amount [26]. It has been shown that after the use of PRP epithelization accelerates along with increased wound tension in a cutaneous incisional wound model [27,28]. This acceleration has been observed in all phases of wound healing, as well as in chronic wounds, especially in diabetic wounds [27,28]. Likewise, many studies have demonstrated that MSCs have beneficial therapeutic effects on wound-healing processes. Most of the properties of MSCs (such as ease of obtaining, expansion capacity, frequent colony-forming properties, differentiation capacity, neoangiogenesis capacity and immunomodulatory effect) obtained from adipose tissue are as favorable as stem cells taken from the bone marrow, and vascularization and flap viability of the skin flap with randomized feeding patterns can be increased after the local administration of MSCs derived from adipose tissue [29,30,31].

The current study demonstrated that corticosteroid administration could reduce the tissue HP level values in a wound while it increased the tissue IL-1β level values. However, although the TNF-α level values and histopathological grade values in the corticosteroid-administered group (Sham-M) were numerically lower than the values of Sham-S and PR groups and quite similar to the values of MC group, corticosteroid could not statistically affect those values in any group. With these results it could be said that the experimental model of this study worked effectively and, similar to the findings in the literature, corticosteroid could suppress wound-healing processes by reducing tissue HP level values and increasing inflammatory cytokine level values [23,24].

In the present study, collagenization grade values were significantly higher in the PR group, compared with the other groups. Unfortunately, the type of the collagen could not be identified in the wound area because of the study limitations mentioned below. In addition, the tissue HP level values obtained from this study also did not support to the collagenization grade values, biochemically. Furthermore, inflammation grade values were significantly higher in the PR group, compared with the other groups. However, in contrast to the literature [27,28], reepithelization grade values were similar among the groups. Moreover, tissue IL-1β and TNF-α level values were similar to the values of Sham-S and MC groups. With these results, it could be said that PRP could not effect to the investigated inflammatory cytokines, biochemically. In addition, unfortunately PRP could not affect the reepithelization of the wound surface. Nevertheless, these findings suggest that PRP could have a therapeutic effect in wound-healing processes and it also could histopathologically improve the wound healing in tissues in which are suppressed by administering the corticosteroid. With these unexpected findings, it is therefore strongly recommended that the therapeutic activity of PRP in suppressed wound healing should be detailed and tested in further studies.

MSCs, however, could not affect histopathological grades, and it was thought this may be caused by the study duration, which was not long enough to allow MSCs to migrate, differentiate in damaged tissue, and reduce the cytokine releasing. When considering biochemical parameters, the tissue HP level value in MC group was found to be much higher than other groups, although the collagenization grade value was not different from the Sham-M and Sham-S groups’ values. Furthermore, the inflammation grade value was found to be lowest in the MC group, although cytokines level values were not different from the Sham-M and Sham-S groups’ values. Similar to PRP, it could be said that MSCs did not reduce the cytokines in the wound-healing process. However, tissue HP level values of MC group suggested that stem cells may have the potential to produce positive therapeutic effects on wound healing. Therefore, it is strongly suggested that the therapeutic effects of MSCs may be demonstrated by further experimental studies conducted with a larger sample size, which examine both early- and late-period findings.

Finally, according to the unexpected results of this preliminary study, it could be considered that PRP and MSCs could remove the biochemical pressure of corticosteroids on wound-healing processes and provide an alternative treatment for non-healing wounds in immunosuppressive and/or corticosteroid-treated patients.

### Limitations

This study has some limitations. First, due to the scheduled study time, the amount of scar formation in the wound was not investigated. Second, due to the scheduled study time, the tensile forces of the skin over the wound were not tested using physical methods at the end of the wound-healing process. Third, detailed histopathological (such as Masson’s Trichrome staining, immunohistochemistry, electron microscopy, fluorescence microscopy) and biochemical analysis methods (such as Western blot, polymerase chain reaction) were not used to demonstrate the curative effect of the experimental biological materials used in this experiment due to technical and financial restrictions. Fourth, this preliminary study included the results of the tissue material analysis on the 10th day of wound healing. Therefore, the biochemical well-being of wound healing by MSCs did not extend to the histopathological data in wounds suppressed by corticosteroids. Accordingly, the effects of MSCs on wound healing should be closely investigated in more detailed, large-scale studies that examine the early and late findings of wound healing and use advanced laboratory methods. Fifth, to observe synergistic effects, this preliminary study could have included a group which consisted of rats given both PRP and MSCs. However, financial restrictions did not allow this idea to be realized. For this reason, it is strongly recommended that this situation be investigated in future studies.

## 5. Conclusions

In conclusion, the results of this preliminary study suggest that MSCs and PRP had positive biochemical and/or histopathological effects on wound healing suppressed by corticosteroid in rats, and these positive effects were more salient in the PR group. Based on these findings, it could be argued that PRP and MSCs are likely to be alternative treatment regimens for non-healing wounds in patients who have been treated with immunosuppressive agents and/or corticosteroids. However, further, large-scale studies that investigate the efficacy of these biological materials are needed to establish a definite conclusion.

## Figures and Tables

**Table 1:** Histopathological grading system which was used to evaluate the wound-healing parameters collagenization, inflammation and reepithelization.

Grade	Collagenization	Inflammation	Reepithelialization
Grade 0	None	None	No epithelization
Grade 1	Minimal	Minimal	Incomplete coverage
Grade 2	Moderate	Moderate	Complete reepithelization
Grade 3	Severe	Severe	-

**Table 2:** Histopathological and biochemical results of all groups.

Group	Variable	Minimum	Maximum	Mean/Median (*)	SD
Sham-S	Collagenization	1	2	2 *	0.46
	Inflammation	1	2	2 *	0.46
	Reepithelization	1	2	1.5 *	0.54
	Hydroxyproline	367.71	1040.57	675.64	220.16
	Interleukin-1β	100.58	138.14	119.89	15.48
	Tumor necrosis factor-α	97.01	134.57	118.75 *	15.48
Sham-M	Collagenization	1	2	1.5 *	0.54
	Inflammation	1	2	1.5 *	0.54
	Reepithelization	1	2	1 *	0.46
	Hydroxyproline	141.89	792.22	439.47	238.99
	Interleukin-1β	53.78	144.07	78.71	27.99
	Tumor necrosis factor-α	17.36	157.57	105.79 *	46.80
PR	Collagenization	2	3	3 *	0.52
	Inflammation	2	3	3 *	0.52
	Reepithelization	1	2	1.5 *	0.54
	Hydroxyproline	696.02	992.97	900.30	89.29
	Interleukin-1β	96.76	129.13	111.66	10.44
	Tumor necrosis factor-α	98.66	131.02	110.30 *	10.52
MC	Collagenization	1	2	1.5 *	0.54
	Inflammation	1	2	1 *	0.52
	Reepithelization	0	2	1 *	0.64
	Hydroxyproline	1044.82	1913.75	1322.80	298.24
	Interleukin-1β	97.78	122.77	108.71	8.11
	Tumor necrosis factor-α	80.26	115.25	99.24 *	10.46

SD, standard deviation; * Median value.

**Table 3:** Statistical differences of the histopathological and biochemical findings among the groups.

Variable	*F*/*X*^2^ (*)	*p*
Collagenization	13.89 *	0.003
Inflammation	14.79 *	0.002
Reepithelization	5.59 *	0.134
Hydroxyproline	22.34	<0.001
Interleukin-1β	8.64	<0.001
Tumor necrosis factor-α	4.71 *	0.194

One Way Analysis of Variance (ANOVA) and Kruskal-Wallis test, *p* < 0.05. *X*^2^, chi-square; F, F score; * *X*^2^ value.

**Table 4:** Binary comparisons of the groups for histopathological and biochemical findings.

Variable	Group (I/J)	MD/Z (*)	*p*
Collagenization	Sham-S/PR	−2.71 *	0.007
	Sham-M/PR	−2.92 *	0.003
	MC/PR	−2.92 *	0.003
Inflammation	Sham-S/PR	−2.71 *	0.007
	Sham-M/PR	−2.92 *	0.003
	MC/PR	−3.06 *	0.002
Hydroxyproline	Sham-S/MC	−647.17	<0.001
	Sham-M/PR	−460.82	0.002
	Sham-M/MC	−883.33	<0.001
Interleukin-1β	Sham-M/Sham-S	−41.17	<0.001
	Sham-M/PR	−32.94	0.004
	Sham-M/MC	−29.99	0.009

Tukey Multiple Comparisons test (*p* < 0.05) and the Mann-Whitney U test with Bonferroni Correction test (*p* < 0.0083). MD, mean differences; *Z*, *Z* score; * *Z* score value.
